# Supplementary figures and images for: Spatial distribution of poultry farms using point pattern modelling: A method to address livestock environmental impacts and disease transmission risks
Source: PLoS Comput Biol. 2024 Oct 1;20(10):e1011980. doi: 10.1371/journal.pcbi.1011980 (PMC11444418; doi:10.1371/journal.pcbi.1011980)

Broiler

Gujarat

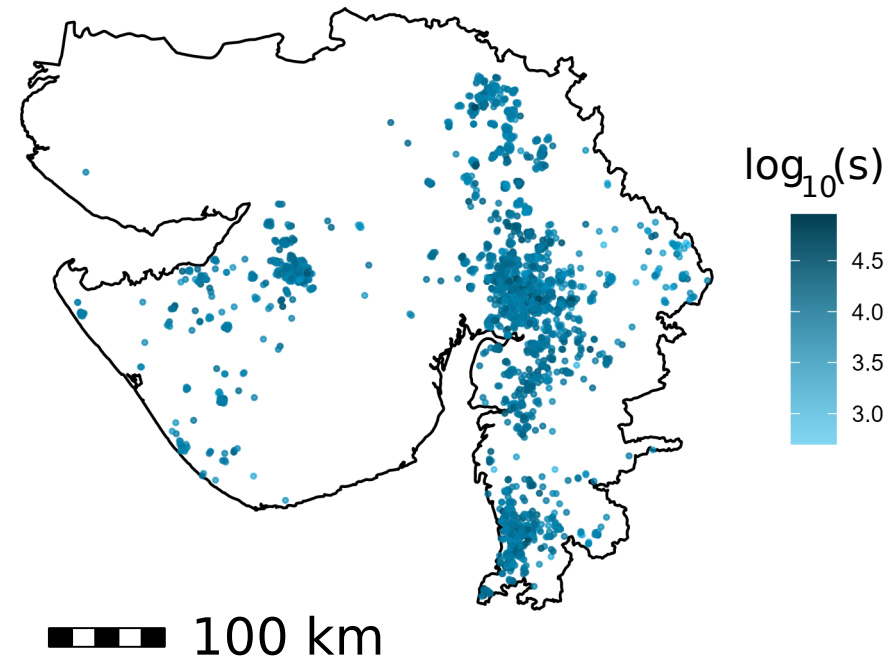

Bangladesh

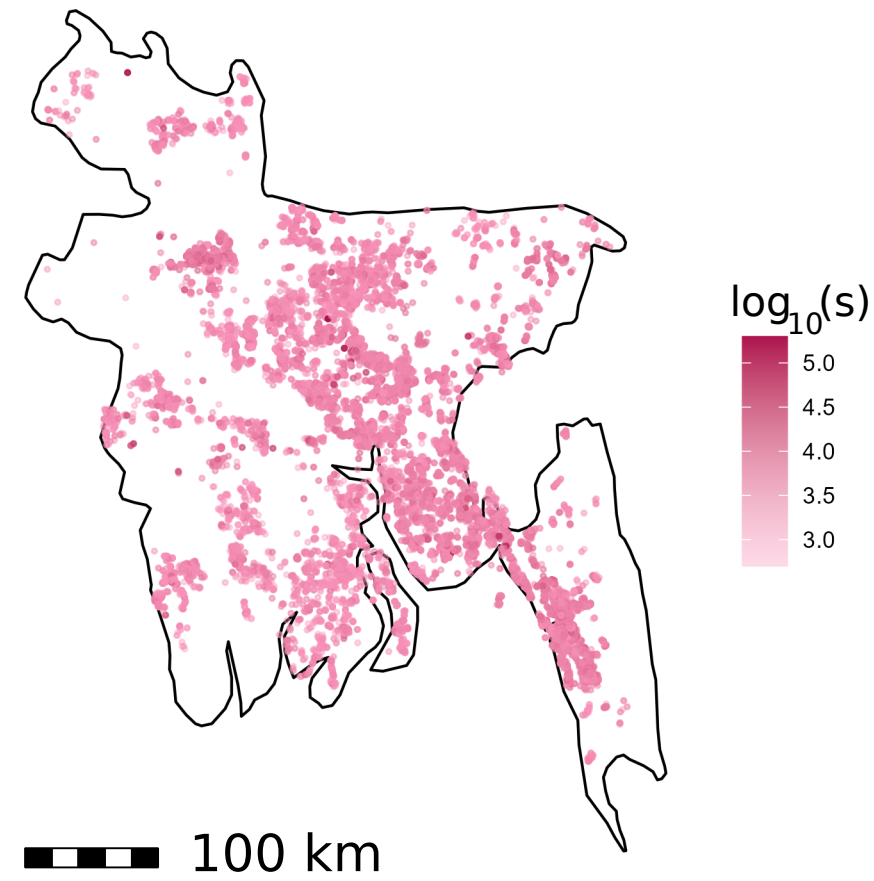

Thailand

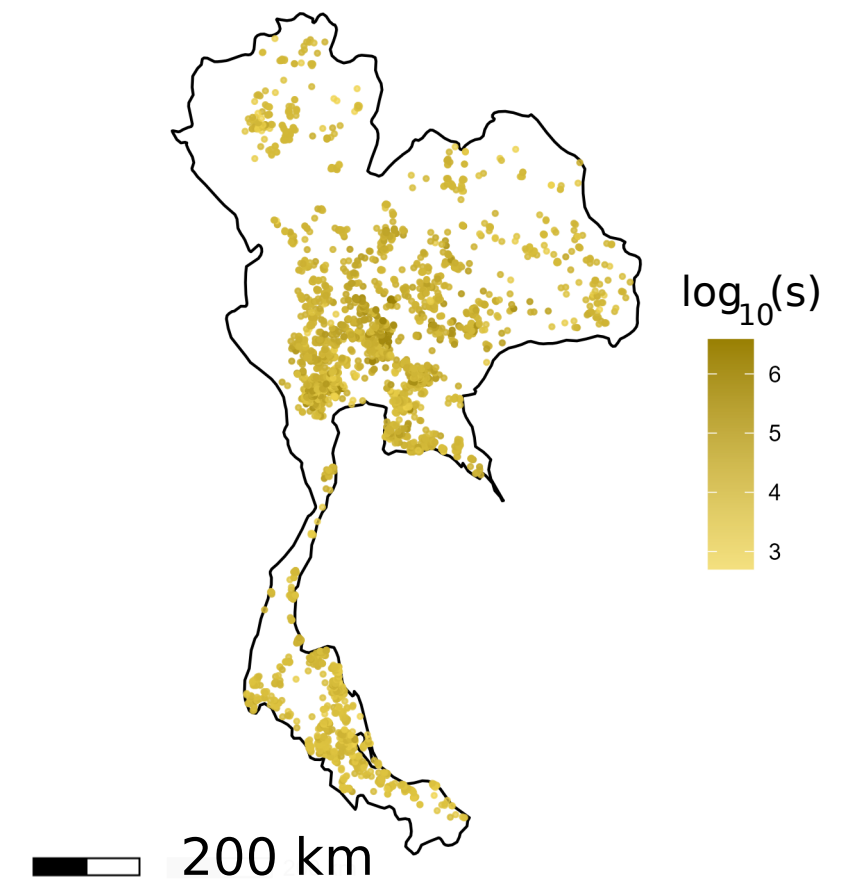

Layer

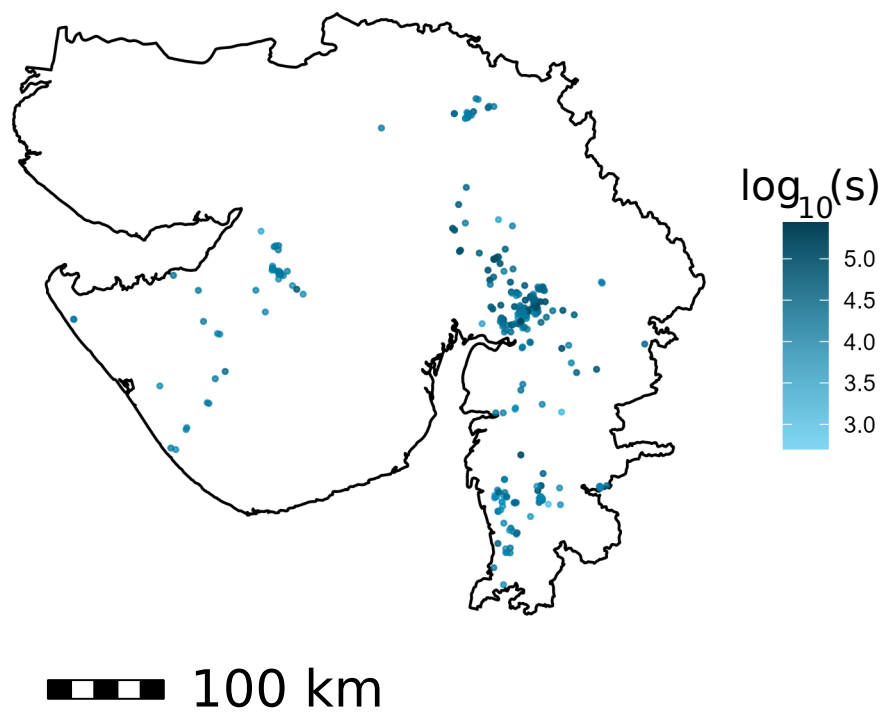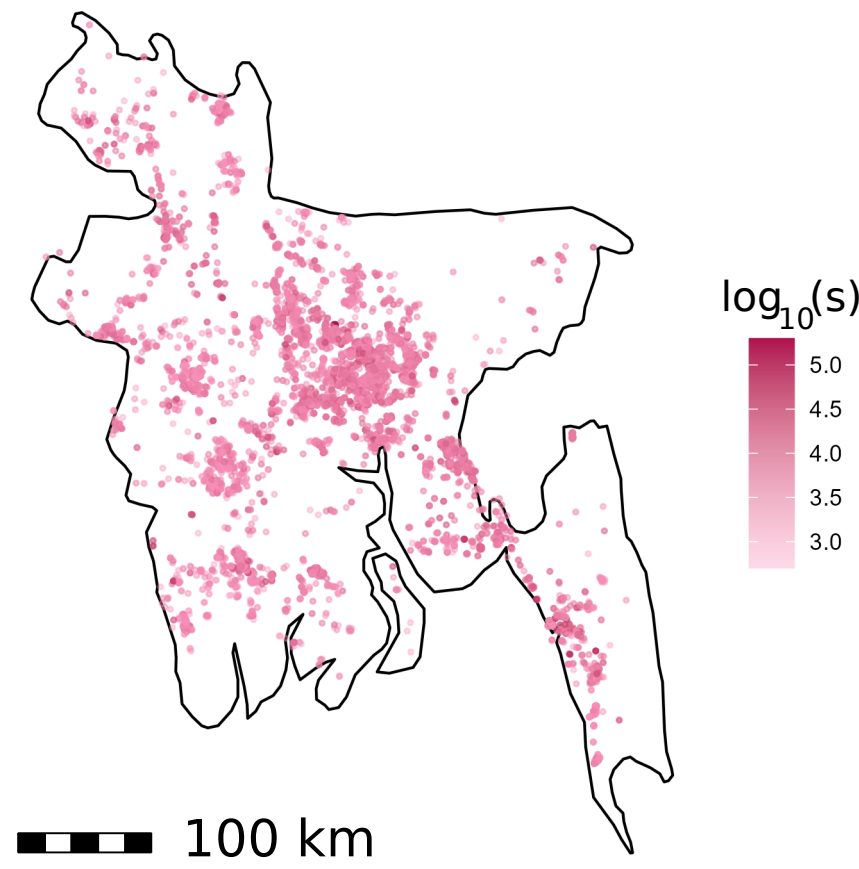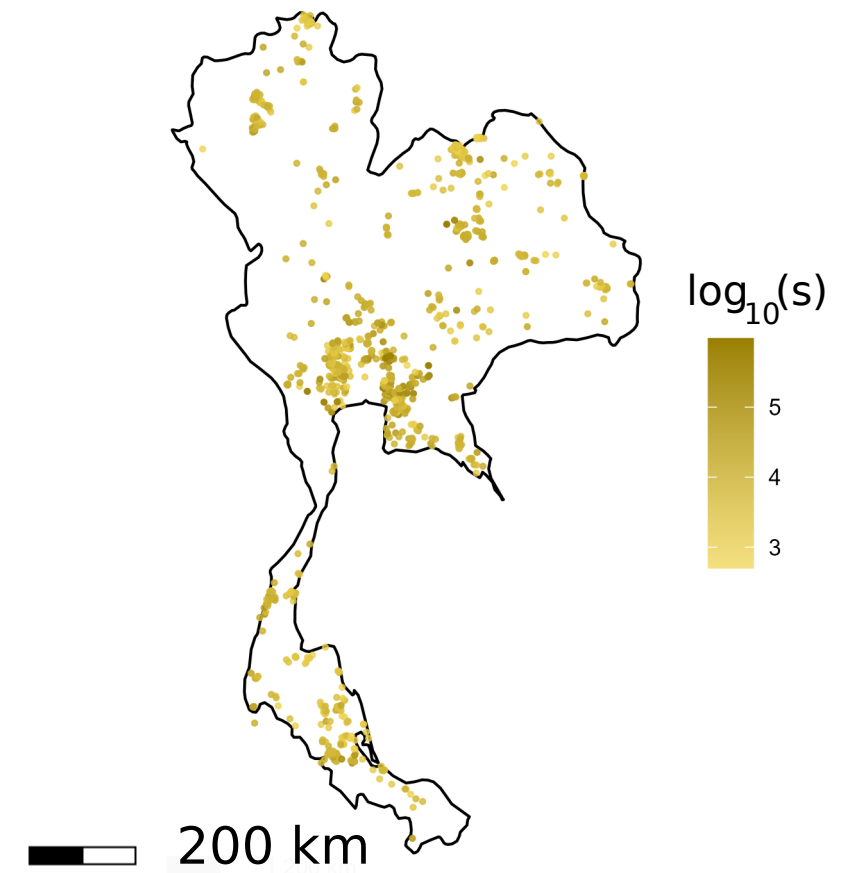

Supplement: S1 Fig — Source of the basemaps: https://gml.noaa.gov/aftp/pub/basu/Borders/GADM/. (PDF) [file pcbi.1011980.s001.pdf]

circle  $b$  of radius  $d$

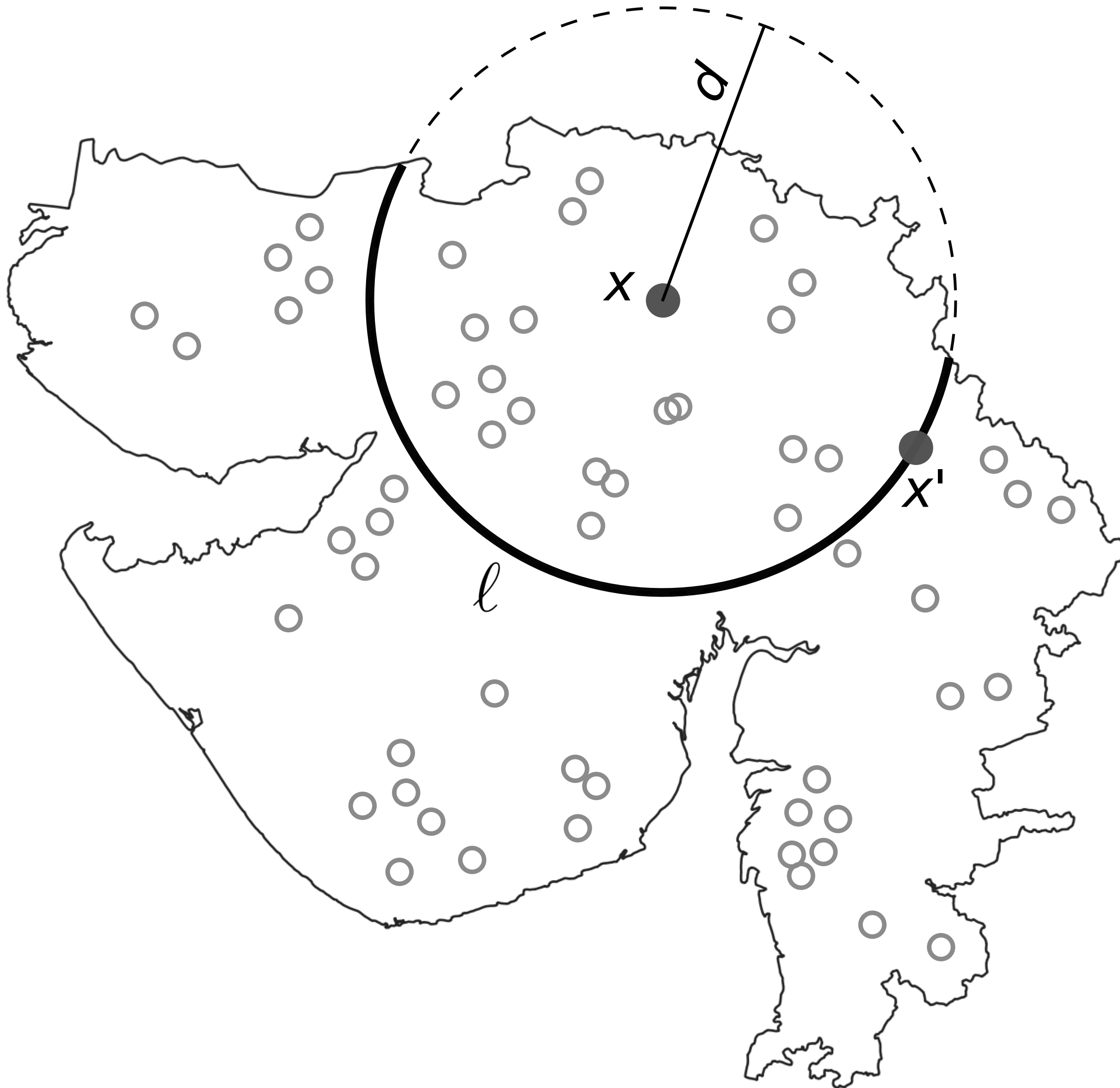

Supplement: S2 Fig — The study area, W, is the administrative border of the study region (here, Gujarat is illustrated). The Euclidean distance d between points x and x′ is shown, along with a circle of radius d centered at x. The portion of the circle, ℓ, inside the study area is highlighted, demonstrating how the edge correction weight eij is calculated. This correction accounts for the fraction of the circle’s length that lies within the study area, ensuring accurate spatial analysis by adjusting for boundary effects. Source of the basemaps: https://gml.noaa.gov/aftp/pub/basu/Borders/GADM/. (PDF) [file pcbi.1011980.s002.pdf]

Gujarat

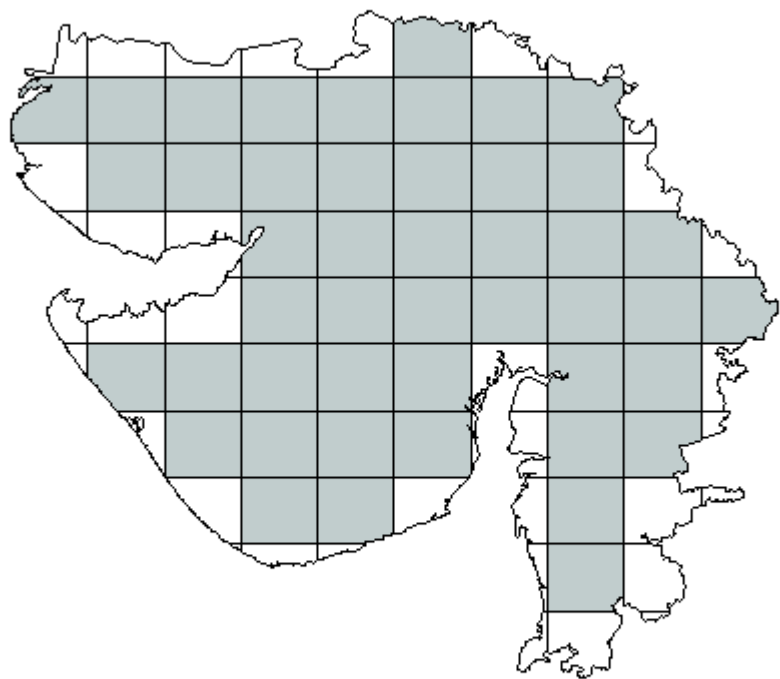

Bangladesh

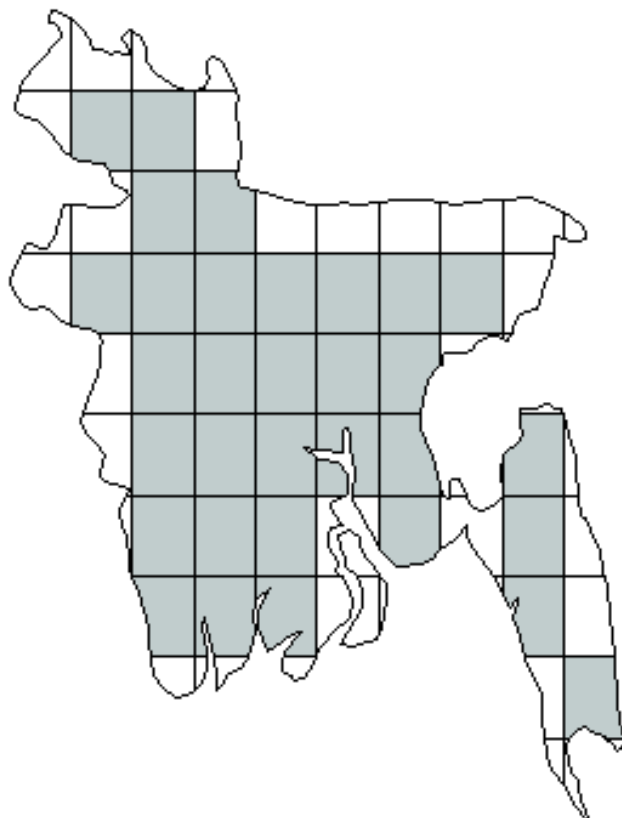

Thailand

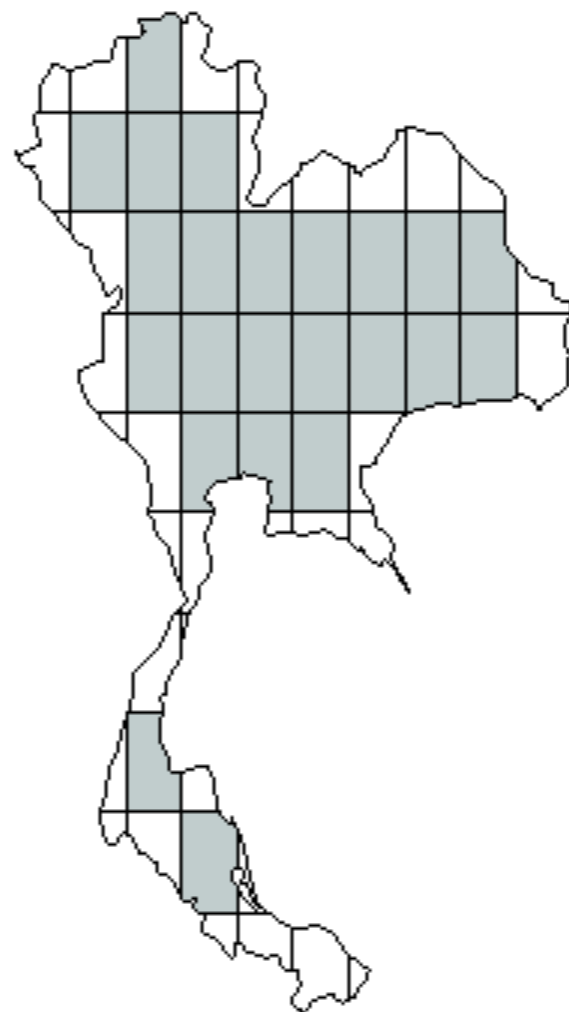

Supplement: S3 Fig — Grey tiles were used to calculate the coefficient correlation between observed and simulated points pattern. We did not consider quadrats that occupy less than 80% of the complete theoretical polygon to avoid edge effects. Source of the basemaps: https://gml.noaa.gov/aftp/pub/basu/Borders/GADM/. (PDF) [file pcbi.1011980.s003.pdf]

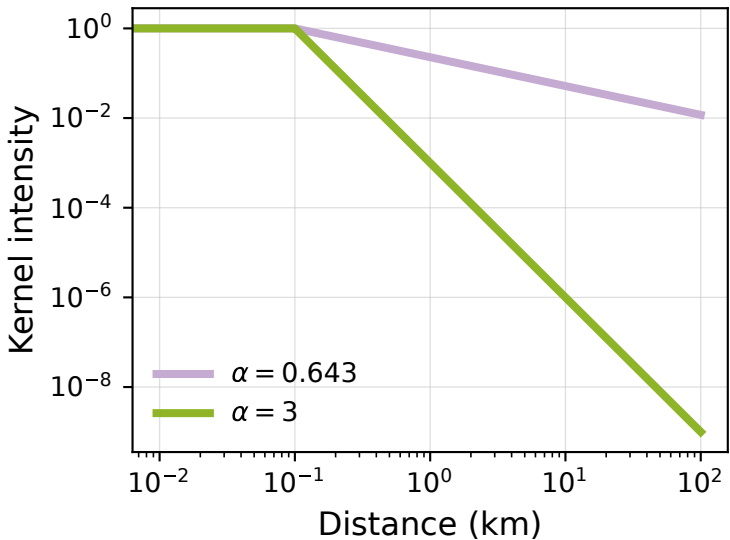

Supplement: S4 Fig — (PDF) [file pcbi.1011980.s004.pdf]

A

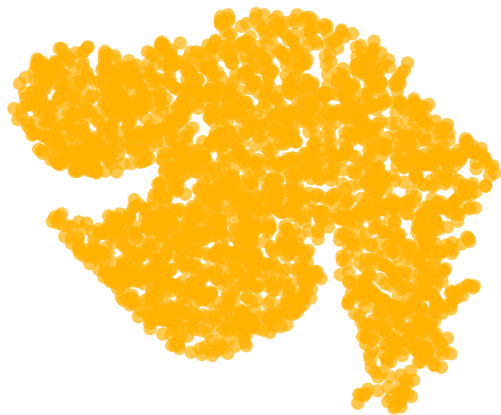

B

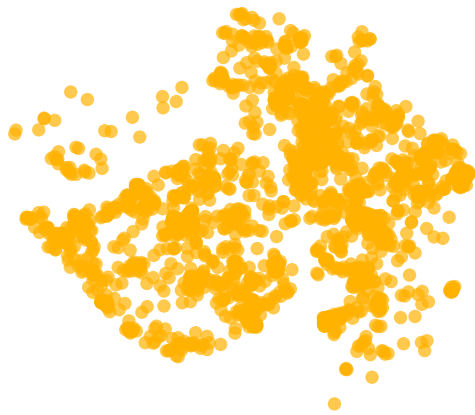

C

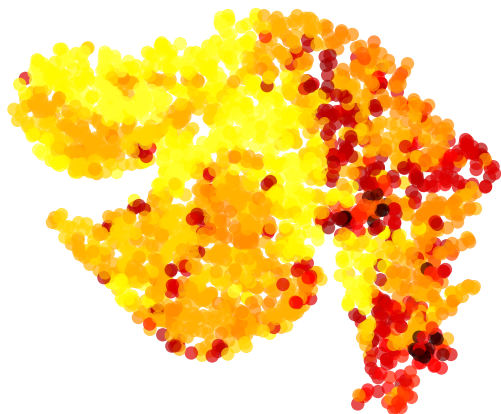

D

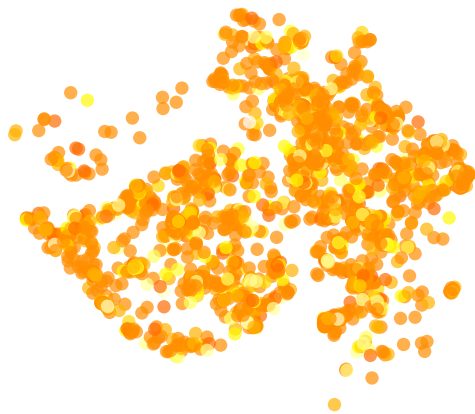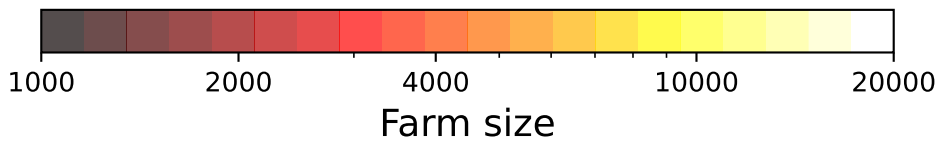

Supplement: S5 Fig — A. Random farms distribution with constant farm size. B. Farms distribution generated with the LGCP model with constant farm size. C. Random farms distribution with farm sizes predicted with RF model. D. Farms distribution generated with the LGCP model with farm sizes predicted with RF model. Source of the basemaps: https://gml.noaa.gov/aftp/pub/basu/Borders/GADM/. (PDF) [file pcbi.1011980.s005.pdf]

A

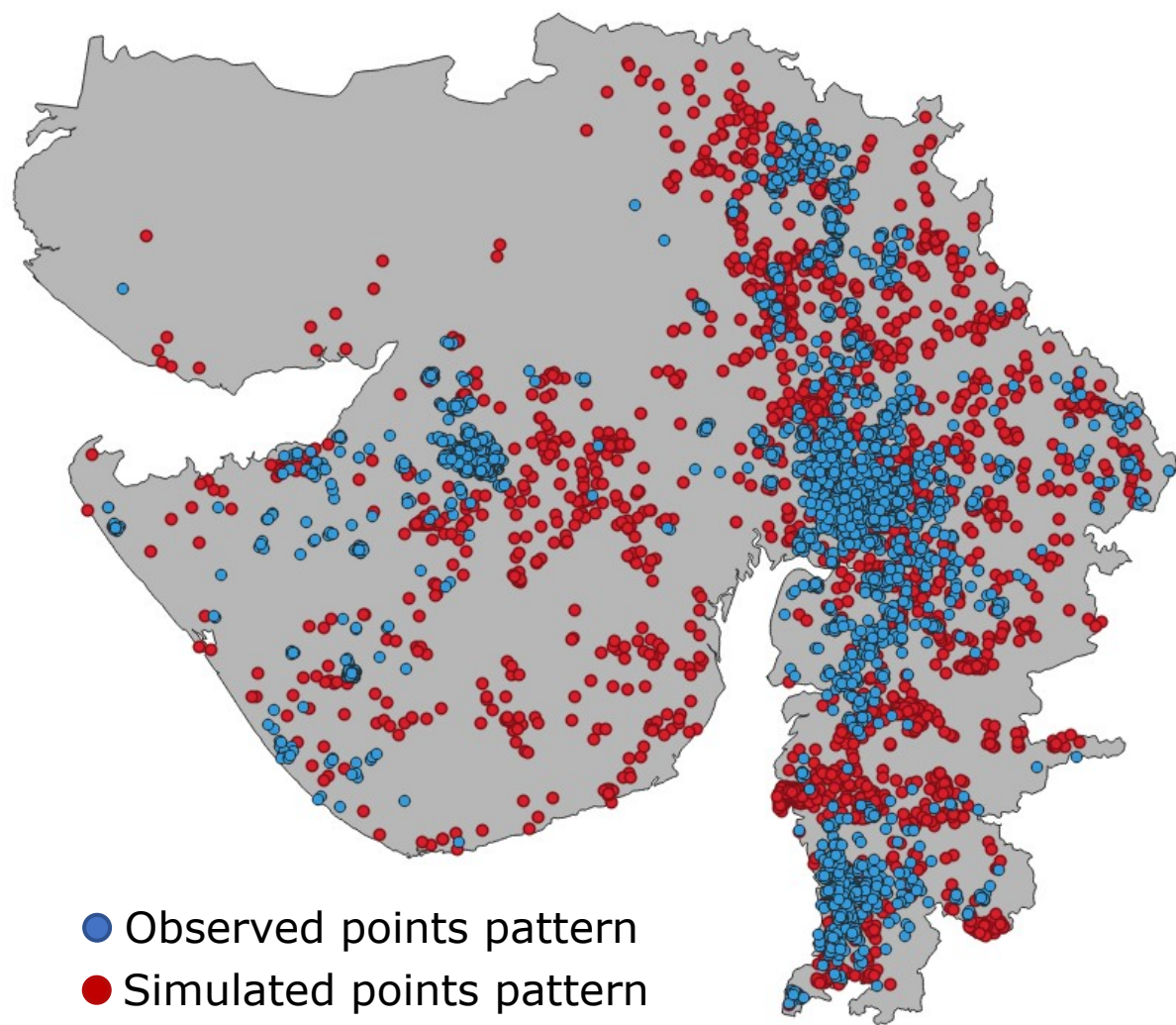

B

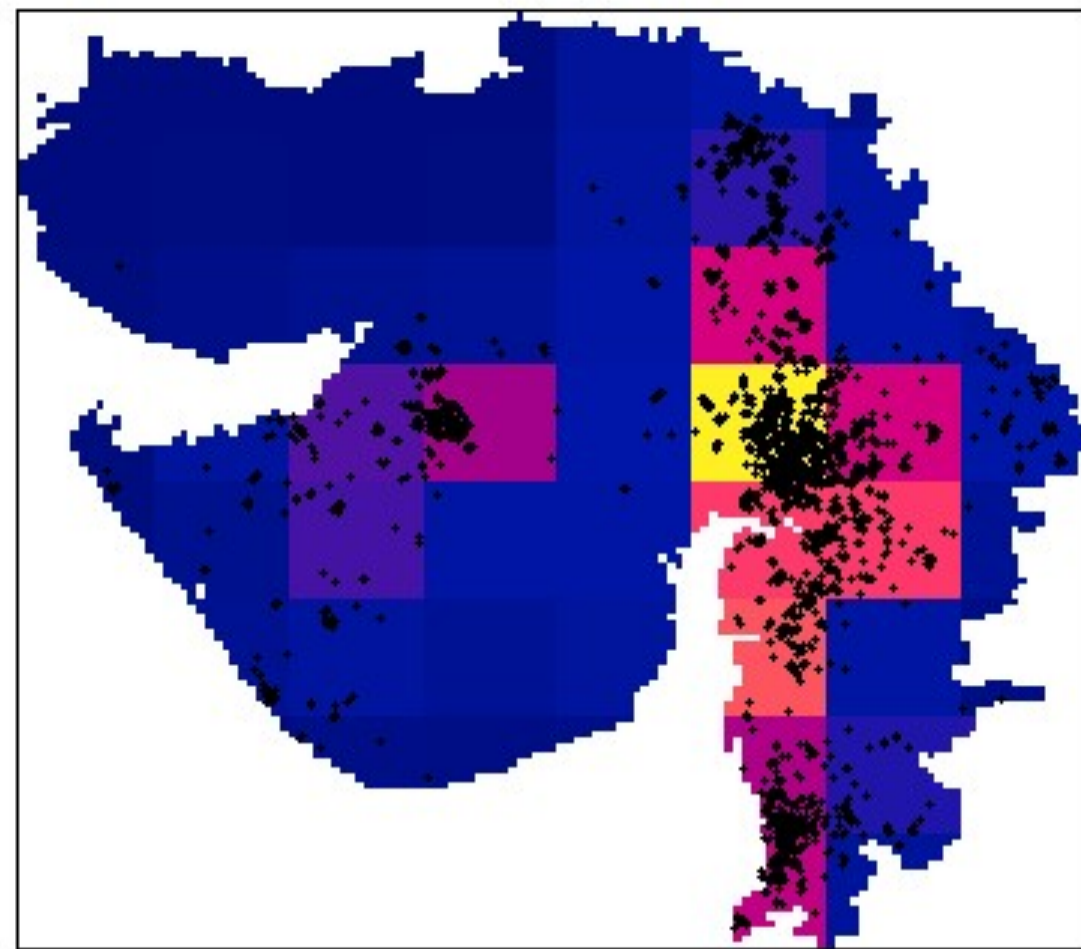

Intensity  
(pts/square unit)

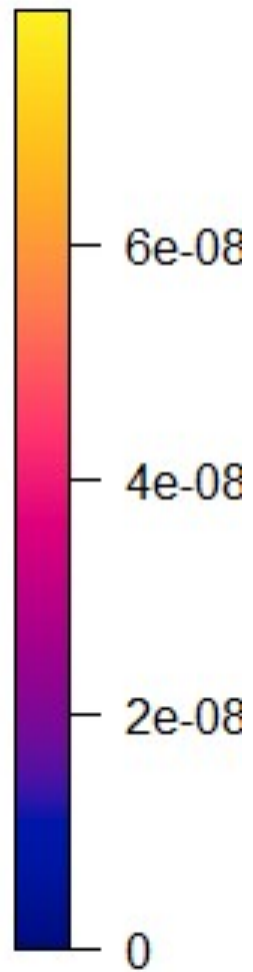

Supplement: S6 Fig — A. Observed points patterns of broiler farms in Gujarat and one simulated point pattern with the model trained with Bangladesh broiler farms. B. Mean intensity of points of 8000 simulations from the model trained in Bangladesh and the observed point pattern is represented with black dots. Source of the basemaps: https://gml.noaa.gov/aftp/pub/basu/Borders/GADM/. (PDF) [file pcbi.1011980.s006.pdf]

## Broiler

## Layer

$r < 20\text{kms}$

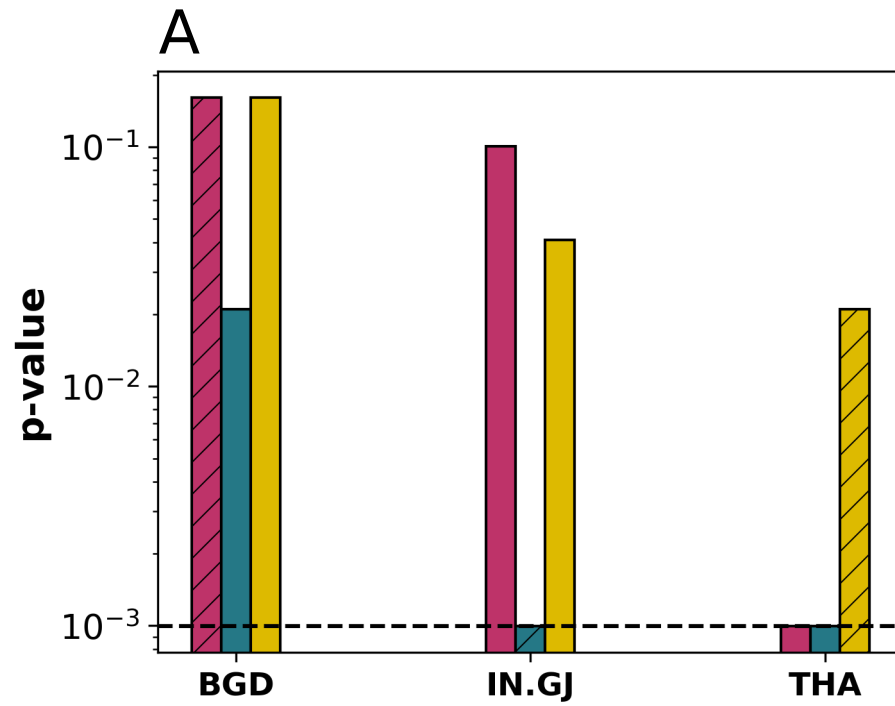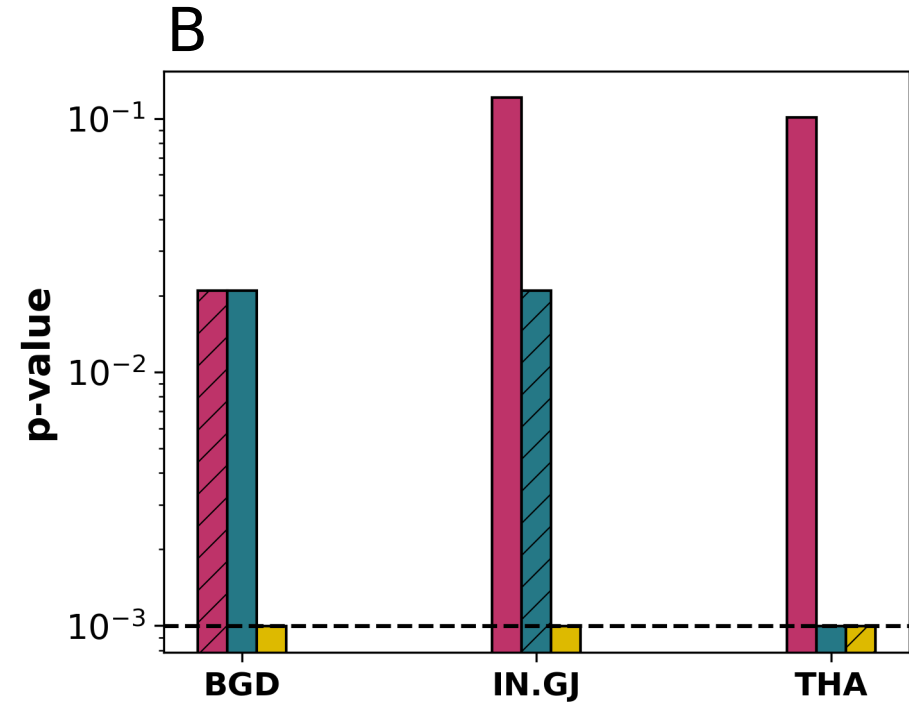

Testing area

**BGD**

**IN.GJ**

**THA**

$r > 20\text{kms}$

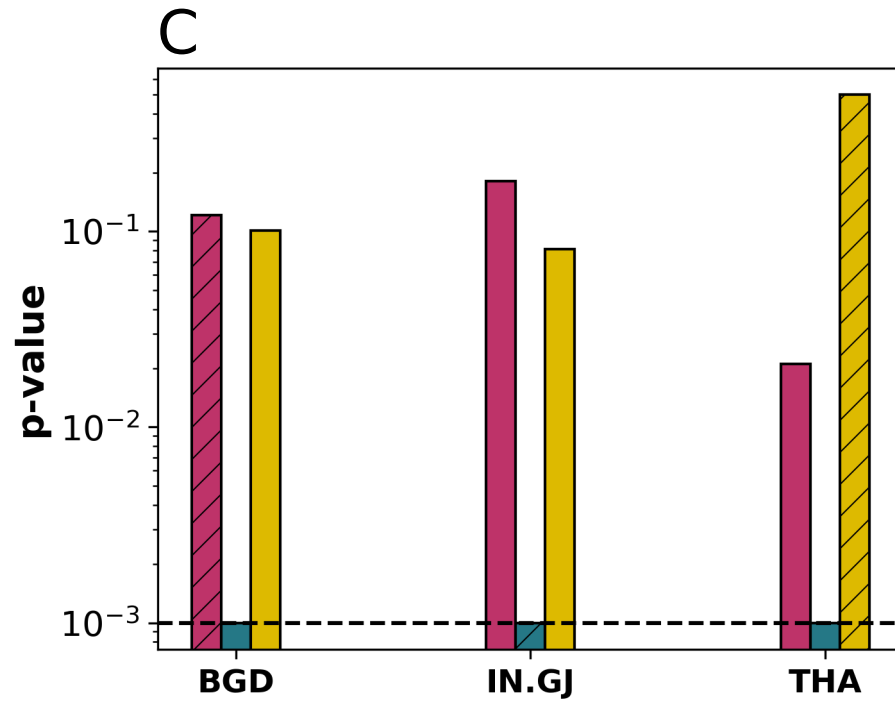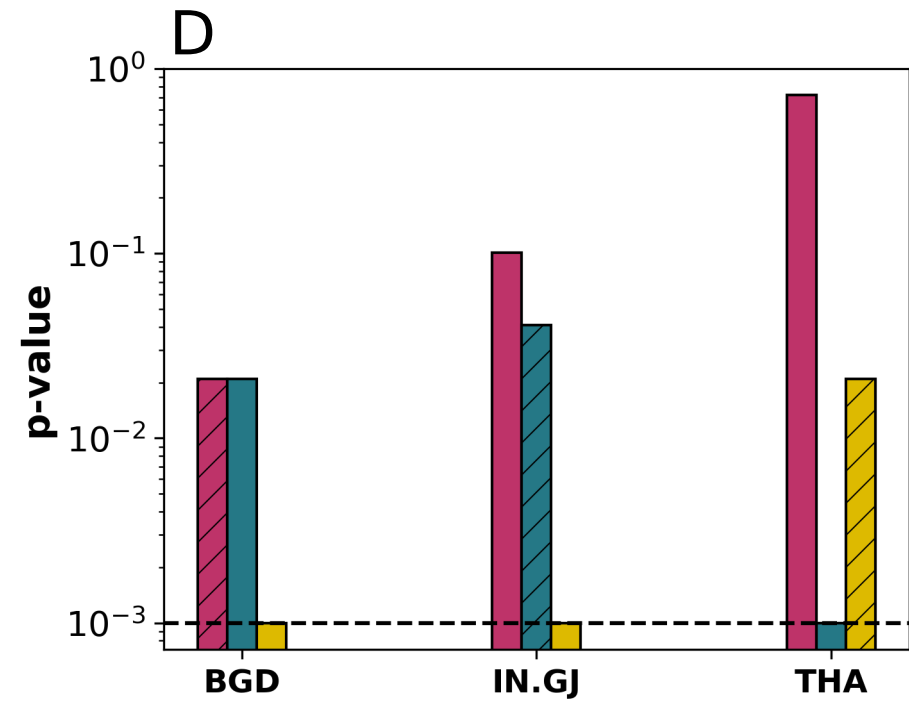

Supplement: S7 Fig — Internal and external validation p-values of the global rank envelope test for the different models (Bangladesh: BGD, Gujarat: IN.GJ and Thailand: THA), for the two types of farm: broilers (A & C) and layers (B & D) and for radii under 20kms (A & B) and for radii above 20kms (C & D). Labels on the x axis denote the training area. Hatched bars distinguish p-values for internal validation from those for external validation. The color of the bar charts indicate where the model is tested (grey for Bangladesh, blue for Gujarat and yellow for Thailand). The horizontal dashed line indicate the threshold of significance of the p-values for the envelope of 1000 simulations. (PDF) [file pcbi.1011980.s007.pdf]

**A**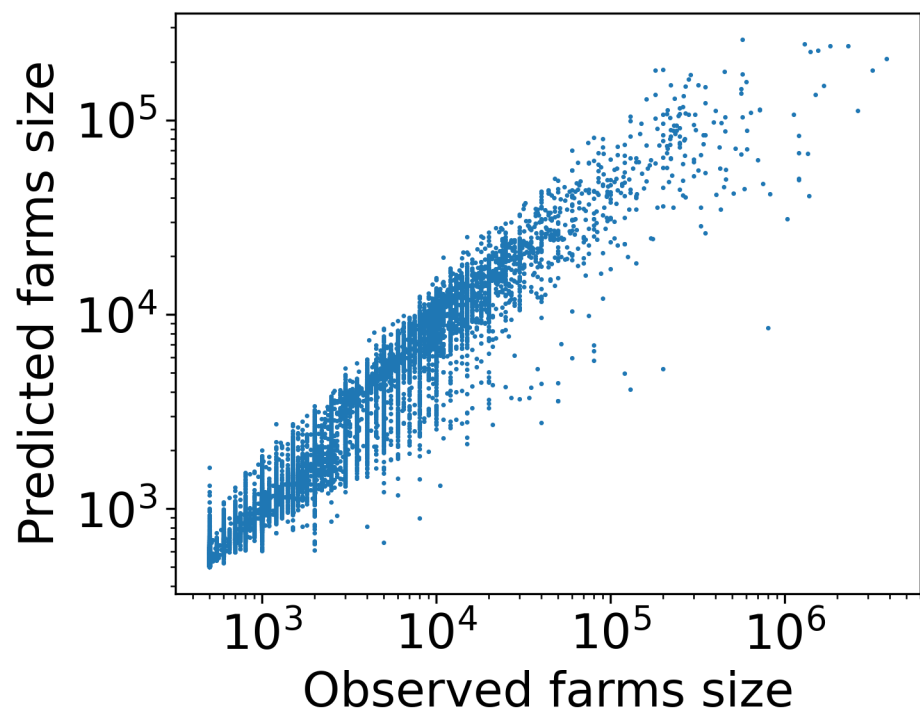**B**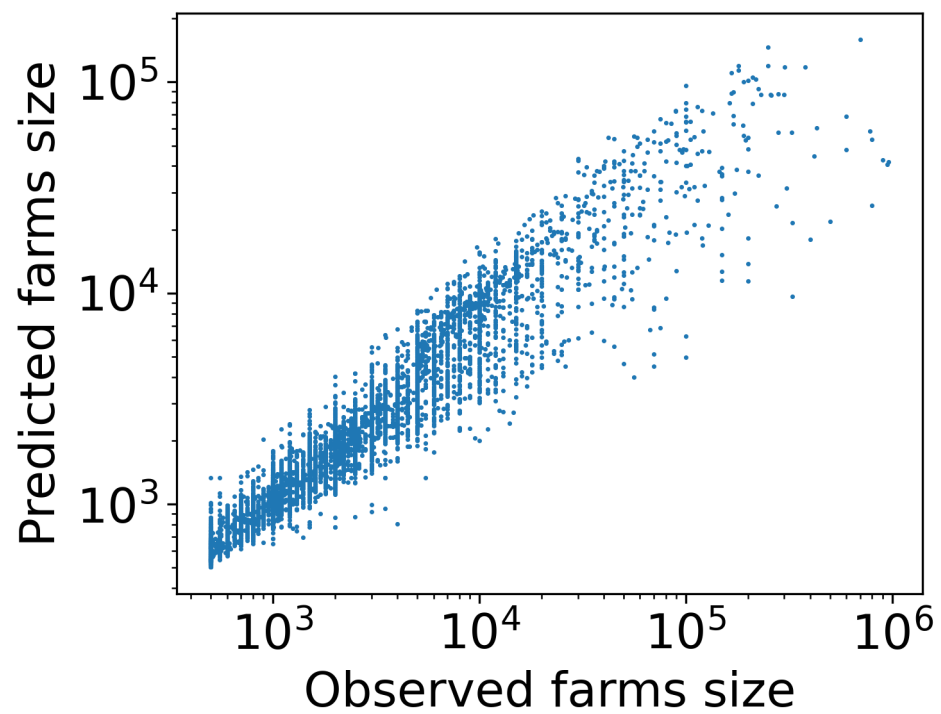**C**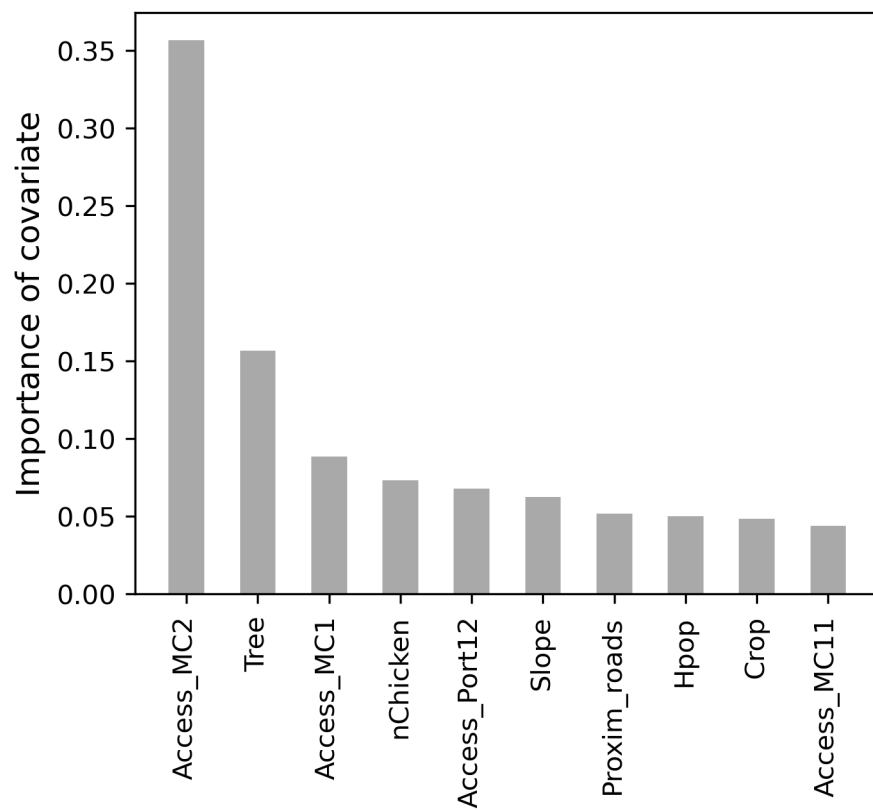**D**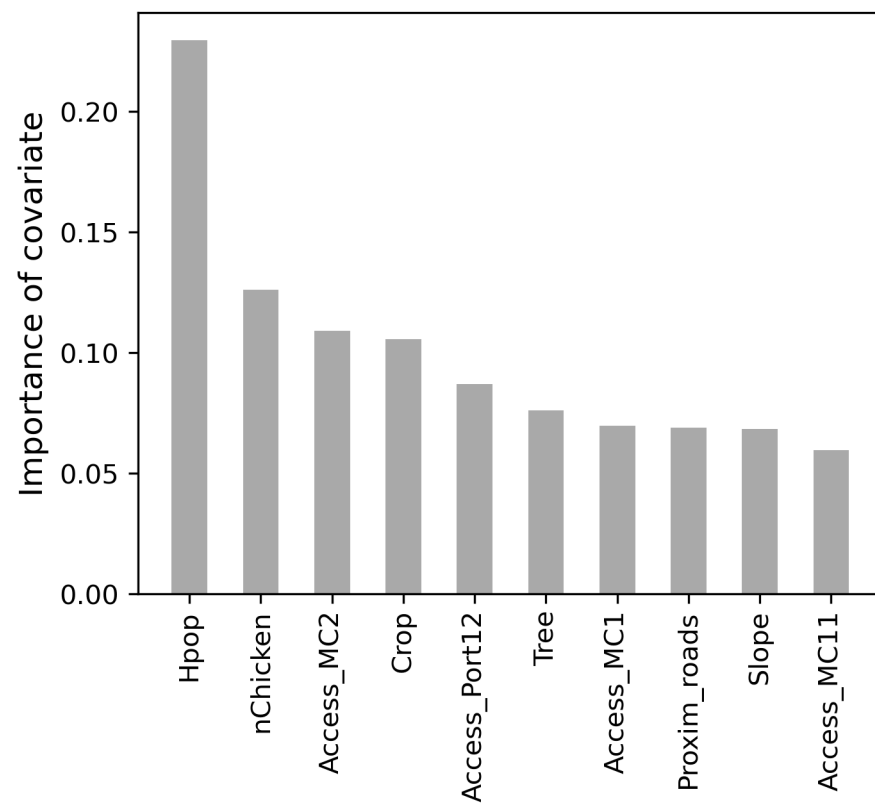

Supplement: S8 Fig — Predicted farm size in function of observed farm size (A. Broiler and B. Layer). C & D. Importance of each covariate for broiler farm RF model (A) and layer farm RF moodel (B). (PDF) [file pcbi.1011980.s008.pdf]

Gujarat

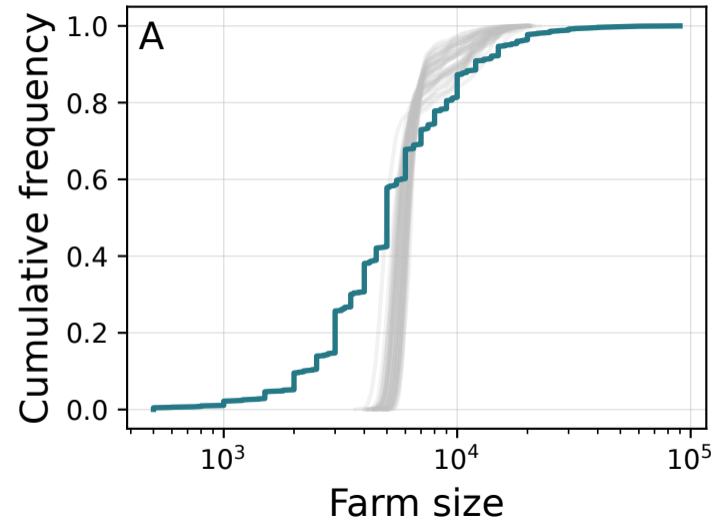

Bangladesh

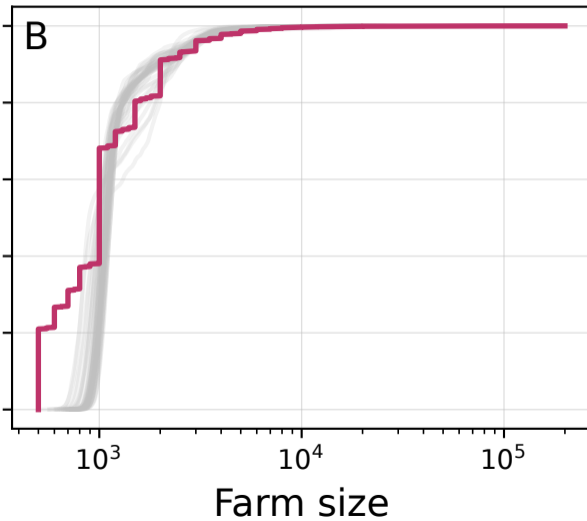

Thailand

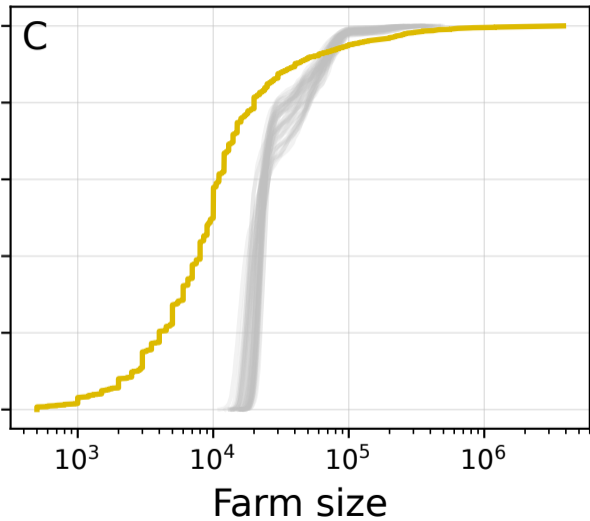

Supplement: S9 Fig — (PDF) [file pcbi.1011980.s009.pdf]

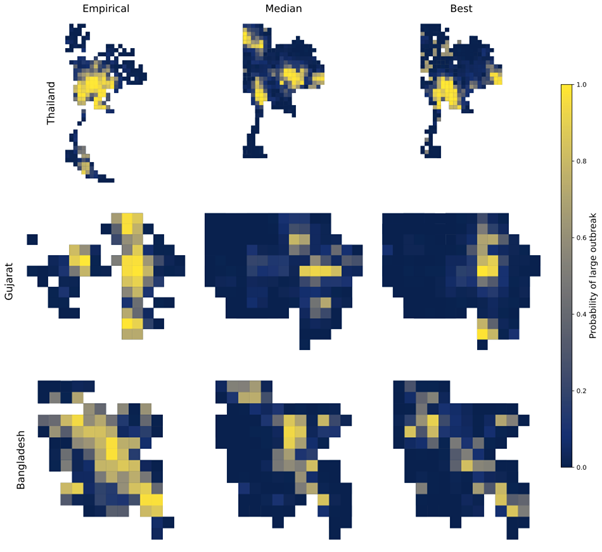

Supplement: S10 Fig — Shown for Thailand (top row), Gujarat (middle row) and Bangladesh (bottom row). All simulations are based on a long-ranged transmission kernel and the middle β values in Fig 7. The first column shows risk calculated from using the empirical farm distribution. The second and third columns use the average- and best-performing point pattern distributions sampled from the iLGCP+RFS model trained on the same area. Performance is based on Spearman’s rank correlation coefficient between gridded risk distributions. White cells contain no farms. Source of the basemaps: https://gml.noaa.gov/aftp/pub/basu/Borders/GADM/. (PNG) [file pcbi.1011980.s010.png]
