# Supplementary material for: Spatial distribution of poultry farms using point pattern modelling: A method to address livestock environmental impacts and disease transmission risks
Source: PLoS Comput Biol. 2024 Oct 1;20(10):e1011980. doi: 10.1371/journal.pcbi.1011980 (PMC11444418; doi:10.1371/journal.pcbi.1011980)
Supplement: S1 File — (DOCX) [file pcbi.1011980.s011.docx]

The test procedure is as follows:

- Generate a large number of simulated point patterns (1000 simulations) based on the fitted model parameters.
- For each simulation and each distance *r*, calculate the test statistic $L_{inhom}(r)$
- Rank the observed test statistic among the simulated values at each *r*.
- Construct the global envelope based on the ranks. In this case, the envelope is constructed using the most extreme ranks from the simulations, i.e. the minimum and maximum (the 1st rank from the bottom and top, respectively) simulated values at each distance *r*.
