# Supplementary material for: Spatial distribution of poultry farms using point pattern modelling: A method to address livestock environmental impacts and disease transmission risks
Source: PLoS Comput Biol. 2024 Oct 1;20(10):e1011980. doi: 10.1371/journal.pcbi.1011980 (PMC11444418; doi:10.1371/journal.pcbi.1011980)
Supplement: Attachment — Submitted filename: Response to reviewer comments.pdf [file pcbi.1011980.s014.pdf]

Response to Reviewers

We would like to sincerely thank the reviewers for their comments, which enabled us to improve the presentation of our results. We have modified the manuscript, and the changes are highlighted in blue in the pdf. We respond to the reviewers' comments point by point in this document. Our responses are also highlighted in blue.

Reviewer #1:

In this manuscript the authors develop a method to predict the location and sizes of poultry farms in country with scarce data.
The manuscript is very well written, the objectives are stated clearly and the method they propose is a very interesting approach to tackle the problem of the scarcity of poultry farms data in some countries.

Thank you very much for your thoughtful and positive review of our manuscript. Your recognition of the method as an interesting approach to address the problem of data scarcity is particularly encouraging to us.

I have only a few comments which I list below:
1) Session 2.3. This is an important part of the method, however the authors don't provide a clear explanation on how they apply LGCP to simulate the spatial distribution of farms. I understand that they use it to simulate an intensity on a certain area u. But what is the grid size of u? I guess they divide each study region in a grid, with each grid pixel being u. But this is just a guess because I cannot find this information anywhere.

Yes, the intensity function is represented as a matrix, so the window is divided into a grid, and each location u is a cell of this grid. We have added more details in this section to clarify the method (lines 147-151): "The spatial distribution of farms was modelled by estimating the intensity function λ(u) on a 128x128 grid, where each grid cell represents a specific spatial location u. This configuration was automatically set by spatstat (version '2.3.3', Baddeley et al (2014)) to effectively capture spatial variations within the study regions (Baddeley et al, 2015)."

2) Section 2.5.1. In the Random Forest model the authors transform farms size data to reduce the skewness. I was wondering why this is necessary. I am not an expert in random forest models, but I know that random forest is a tree-based model and as such it does not requires scaling of the data.

In the Random Forest model, scaling the target variable (such as normalising between 0 and 1 by the maximum value) isn't necessary because tree-based models don't depend on the scale of the data. However, transforming skewed data to a more normal distribution, through a log transformation, can help improve model performance. A more normalised distribution helps to make more balanced splitting decisions between trees in the forest, which can improve model generalisation.

Therefore, while scaling data is not needed, transforming skewed data is a methodological choice to optimise model performance and ensure more reliable predictions.

Reviewer #2: Title: Spatial distribution of poultry farms using point pattern modelling: a method to address livestock environmental impacts and disease transmission risks

This paper speaks to the need for livestock production metrics and the challenge in producing such metrics where data is limited. To address this challenge, the authors develop a (or build upon Chaiban 2021's) farm distribution model to predict the location and size of poultry farms and test it using farm data from three regions over varying levels of intensification– Bangladesh, Gujarat (a state in India), and Thailand. They use a two-pronged approach to develop the models – first simulating farm locations in point process models and second simulating farm sizes using random forest models. To show the relevance of identifying farm clusters and sizes, they ran simulations of pathogen transmission across different spatial patterns and showed that their distribution model aligned with simulations produced from observed data. This work is important and advances the scientific ability to build higher resolution and more reliable risk analyses that currently use polygon (county, state, etc) level poultry density estimates (such as provided by Van Boeckel 2012, or GLW). The authors' results show that the non-homogeneity in spatial distribution of farms (and associated farming intensity, or number of animals in production within a farm) is an important component in ensuing risk models. The paper is generally well written, however, I have outlined a number of minor detailed comments that should be addressed below.

Thank you very much for your comprehensive and insightful review of our manuscript. We greatly appreciate your recognition of the importance of livestock production metrics and the challenges associated with limited data availability. We are pleased to hear that you found our approach, which builds upon Chaiban (2021), valuable in advancing the prediction of poultry farm locations and sizes.

We have taken into account the detailed comments you have outlined and have made the necessary revisions to enhance the clarity and quality of our manuscript. We believe these changes address your concerns and further strengthen our work.

Detailed Comments:

1. For future efforts, please provide line numbers within your document to ease the communications in the review process.

We have added the line numbers to the document.

2. Page 2, 1st paragraph in Introduction – "In Asia, chicken meat production has quadrupled in the last two decades (FAOSTAT);". FAOSTAT reference is missing from Lit Cited section.

We have added the reference in the list.

3. Page 2, 1st paragraph in Introduction ; "this rapid intensification has been characterised by geographic displacement of farms,". How has rapid intensification of farming led to geographic displacement of farms? Did original farms close down and move to less rural areas?

The turnover of farms may be high; rural farms may close down rather than relocate, with new farms opening in peri-urban areas. The use of the term "displacement" may be misleading, so the sentence has been amended as follows: "This rapid intensification has been characterised by a shift in the spatial distribution of farms, particularly pig and poultry farms which have smaller land requirements than ruminants, transitioning from rural to peri-urban areas." (lines 40-44).

4. Page 2, 2nd paragraph: "However, many LMICs do not have the resources needed to keep track of exact farm locations." These issues also exist for non-LMICs because of privacy issues.

We agree with this comment and have therefore amended the sentence to read: "However, recording exact farm locations may not be achievable in many LMICs due to limited available resources and may also be constrained for privacy reasons, including in HICs." (lines 47-49).

5. References:
Fix formatting of refs, for example:

UK should be capitalized : Keeling, M.J., Woolhouse, M.E., Shaw, D.J., Matthews, L., Chase-Topping,
M., Haydon, D.T., Cornell, S.J., Kappey, J., Wilesmith, J., Grenfell, B.T.,
2001. Dynamics of the 2001 uk foot and mouth epidemic: stochastic dispersal
in a heterogeneous landscape. Science 294, 813–817.

Not full reference: Steinfeld, H., 2006. Livestock's long shadow: environmental issues and options. Food & Agriculture Org.

We have corrected the formatting of the references.

6. Page 3, "However, the distribution of animals at farm level and the process of generating clustered point distributions are yet to be embedded in these models." This has been done for some regions. For example, see Patyk KA, McCool-Eye MJ, South DD, Burdett CL, Maroney SA, Fox A, Kuiper G, Magzamen S. Modelling the domestic poultry population in the United States: A novel approach leveraging remote sensing and synthetic data methods. Geospat Health. 2020 Dec 10;15(2). doi: 10.4081/gh.2020.913. PMID: 33461269.

Thank you for the reference, we have added it lines 62-65 page 3.

7. Table 1. The GDP metric is noted for 2019. The text above the table says that data is for 2020. Is it only GDP that is 2019? You could clarify in the title of the table that data is for 2020.

Thank you for pointing out the need for clarification in Table 1 regarding the year of the data. The GDP metric is indeed noted for 2019, while the other data is from 2020, 2010, and 2015 as specified. We have clarified this information in the description of the table.

8. Table 2. Title "List of spatial predictors". Consider having a more complete title.

Thank you for your suggestion regarding Table 2. We have updated the title to be more comprehensive and informative.

9. Page 6, The inhomogeneous K-function… "where N is the number of points, |W| denotes total study area, I{dij ≤ r} equals 1 if the euclidian distance dij between points i,j is less than r and is 0 otherwise, and eij is an edge correction weight to avoid sampling biases. Given the location of the first point x and the distance d = ||x − x′||, the second point x′ must lie in the circle b of radius d and centred at x. However, the circle b is generally only partly inside the study area W for large d. Then, the Ripley's isotropic correction eij uses the fraction of the length of the circle, ℓ, that is ithin the study area and considers that the point pattern is isotropic (statistically invariant under rotation). We calculated the probability of the second point x′ being inside the window W as"… Consider having a supplemental that draws out an example of the spatial application of these equations.

Thank you for your feedback regarding the explanation of the inhomogeneous K-function and edge correction. In response to your suggestion, we have included a supplemental figure (Figure S2) that visually illustrates an example of the spatial application of these equations.

10. Page 7, Global rank envelope test. Please add references for the tests that you used. Similarly, add the R/Python packages used. Indeed, this can be found in your github link for your code, but it would be nice to have it in the main text, as well. It's listed for some of your methods, but not all.

We have added the relevant references for the statistical tests and specified the R packages and functions used in the main text of the manuscript (lines 195-196): "The test was performed using the `envelope` and `Linhom` functions from the `spatstat` package in R (Baddeley et al., 2015, 2014b) ".

11. Page 8, Section 2.6.1. What determines a removed farm (R)? Does this mean that a farm had an infection and that once this happens, the model does not allow it to become infected again? Can you please explain this action and the thought process underlying it?

Thank you for your inquiry regarding the definition of a removed farm (R) and the underlying thought process. We have added further details on this topic in lines 245-251:

"At the end of the infectious period, the removed state describes premises that were culled following reporting of disease and/or anomalous excess deaths caused by the viral incursion

(which we are not modelling explicitly). We assume that these farms are depopulated and remain empty until the end of the outbreak. Therefore, removed farms are not infectious and can not be reinfected in simulations. These assumptions are more appropriate to describe the spread of a highly pathogenic avian influenza virus strain such as H5N1."

12. Page 9, Section 2.6.2. Can you give more information here on the applicability of the long and short range transmission kernels? Confirming that the alphas were determined by Hill 2017.

The exponent alpha for the long-ranged kernel was parameterised as in Table 2 in Hill et al, 2017 (please note that their notation is different, but the values we use are consistent with their estimates). This value was obtained in the context of a H5N1 AIV outbreak in Bangladesh. We considered a larger value of alpha (α = 3) mainly for theoretical reasons, to explore hypothetical scenarios where AIV spreads preferentially between neighbouring farms. Short-ranged kernels are also relevant to model inter-farm transmission of AIV, as relatively large values of alpha have been already observed in previous studies (Lambert 2023, https://link.springer.com/article/10.1186/s13567-023-01219-0).

We have added details on the manuscript (lines 266-269):
“The former value was retrieved from Hill et al. (2017), while the latter is compatible with shorter transmission lengths across farms as observed in earlier works (Lambert et al., 2023).”

13. Figure 1. The description in the title for Panel A is helpful.

We have added titles of the panels in Figure 1.

14. Page 12, “Crop and human density are important for all models.” According to Fig. 1B, Crop appears as important as the 4 following variables (Access_...) which are the least important except for slope, according to the legend.

Yes, we agree with the comment, and we changed the text accordingly lines 320-322: “Human density is important for all models, while other accessibility predictors, crop and slope were the least important covariates.”.

15. Fig. 2 title “Envelope test of simulated SPPs generated with the model trained in Bangladesh, in respectively Bangladesh, Thailand and Gujarat.” Please reword this sentence and the one following, as they do not make sense. Also note in the title that the grey shaded areas are the envelope.

We have reworded the legend to enhance clarity: “Envelope test of simulated SPPs generated with the model trained in Bangladesh, Applied in Bangladesh, Thailand, and Gujarat. ”.

16. Page 12, Section 3.2.1. “For broiler farms, the model trained using Bangladesh data offers the best prediction in terms of both internal and external validation. Indeed, the envelopes generated with the model trained on Bangladesh data and applied to Bangladesh and Gujarat include, or are near, the respective observed L-function (Figure 2). Although the Bangladesh model underestimates the clustering level of the Thailand SPP (2B), it

reproduces the L-function for low radii of the Gujarat SPPs (Figure 2C) even though it is different from the Bangladesh L-function. Moreover, the Bangladesh-trained model locates the cluster better with high correlation coefficient between observed and simulated SPPs (Figure 3C)". I follow parts of this section (ie, tighter correlation for models trained on their own data). I do not follow what you are saying for Fig. 3C, nor do I see a C panel in Figure 3. Similar comment through Page 13. Please revise and clarify these sections.
We have corrected the numbering of the figures, line 344 and line 354.

17. Page 16 – similar comment as the preceding comment – I do not see a Panel D for Figure 3.
We have corrected the numbering of the figures, line 371 and line 372.

18. Figure 6. To help the reader more quickly associate the 3 locations, consider outlining the plot borders with the color represented for each location in the other figures (grey, blue, yellow for BGD, GJ, THA, respectively). If you do so, I'd suggest changing the blue color that's in Fig 4 and others that represent testing data to one that is different than that used for GJ in Fig 3 and elsewhere.

Thank you for your suggestion regarding the use of colours for outlining plot borders in Figures 6 and 7. We decided not to change the colours of the plot borders for aesthetic reasons, as it could potentially detract from the overall visual balance of the figure. However, we have updated the blue colour in Figures 3 and 4 to a darker shade to ensure it is easily distinguishable from the colour used for Gujarat.

Additionally, to maintain consistency across all figures, we have standardised the colours for each location as follows: Thailand is now represented in yellow, Gujarat in blue, and Bangladesh in red-pink. This change should help readers quickly and clearly associate each location with the corresponding colour throughout the figures.

19. Figure 7. Comment for Fig 6 can also be applied here.

20. Page 26, Final paragraph of conclusions: "Direct applications of our study include the testing of different levels of clustering and the assessment of their impacts on epidemic spread (Benincà et al., 2020). By manipulating the degree of clustering in farm distributions and the clusters locations, policy-makers and stakeholders can gain insights into the vulnerability of livestock systems to disease outbreaks. Understanding the relationship between clustering and epidemic spread is crucial for transitioning towards safer production systems." It is unlikely that decision makers will have the capacity/time to test and manipulate the different degrees of clustering. Are you providing your model as a tool for them to use to do so? This needs to be clarified, if so. The preceding paragraph reads to be the overarching conclusion, but please adjust this paragraph to clarify and strengthen your conclusions.

Indeed, we cannot expect policy makers to use the model, but instead the outputs. We have therefore amended the paragraph as follows (lines 573-580):

“Direct applications of our study include the testing of different levels of clustering and the assessment of their impacts on epidemic spread (Benincà et al., 2020). By manipulating the degree of clustering in farm distributions and the clusters locations, the vulnerability of livestock systems to disease outbreaks can be explored. Generating such an understanding of the relationship between clustering and epidemic spread is crucial to support policy makers and stakeholders in planning the transition towards safer production systems.”.
